# The Effect of Iron Dextran Injection on Daily Weight Gain and Haemoglobin Values in Whole Milk Fed Calves

**DOI:** 10.3390/ani10050853

**Published:** 2020-05-14

**Authors:** Jennifer Allan, Peter Plate, Steven Van Winden

**Affiliations:** 1 The Royal Veterinary College, Regional Veterinary Centre South of England, Stinsford Business Centre, Kingston Maurward College, Dorchester DT2 8PY, UK; pplate@rvc.ac.uk; 2 Department of Pathobiology and Population Sciences, The Royal Veterinary College, Hawkshead Lane, Hatfield, Hertfordshire AL9 7TA, UK; svwinden@rvc.ac.uk

**Keywords:** anaemia, haemoglobin, iron, whole milk

## Abstract

**Simple Summary:**

Whole milk contains low levels of iron, as such this trial examined iron deficiency anaemia in dairy calves fed whole milk. Our trial evaluated the effect of iron supplementation on growth rate and haemoglobin (Hb) levels of these calves. We enrolled 237 calves across six farms, each farm had half of its calves injected with iron and half without. Calves were weighed three times throughout the study and had haemoglobin levels measured at one and six weeks old. Iron caused an average increase in growth rate of 78 g/d (Standard Deviation (SD) 18 g/d) in injected calves compared with control calves. Iron caused a significant increase of haemoglobin levels by six weeks, and haemoglobin levels in the calves that had not received the iron, the iron dropped by an average of 12.1 g/L (SD 15.1). Calves with a higher growth rate in the first six weeks were also more likely to have low Hb levels at six weeks. There was variation in the growth rate differences and haemoglobin levels on each farm. However, overall across all farms, iron makes a difference to daily weight gain (DG) in the first six weeks but the magnitude of effect seems to be farm-specific, as there is a notable difference between farm DG variations.

**Abstract:**

Anaemia caused by iron deficiency has long been reported in dairy calves. This study investigated iron deficiency anaemia on UK dairy farms feeding whole milk and evaluated the effect of iron supplementation on the daily weight gain (DG) and haemoglobin (Hb) levels of these calves. Two-hundred-and-thirty-seven calves were enrolled across six farms. At enrolment, calves were randomly allocated to either receive treatment with iron injection (INJ; n = 120) consisting of 5 mL (1 g iron) of iron dextran (Uniferon 20% Injection, Pharmacosmos) or no injection, control (CON; n = 117). Calves were blood-sampled for Hb and total proteins and weighed at weeks one, six and 12 of age. Iron had a significant effect on DG from one to six weeks, with an average 78 g/d (SD 18 g/d, n = 188, 95% Confidence interval: 44–112 g/d, *p* < 0.001) DG increase in the INJ calves. Iron had a significant effect on Hb concentration at six weeks between the INJ group and CON group (110.7 (SD 12.4) versus 94.9 g/L (SD 13.2), respectively). Calves with a higher growth rate from one to six weeks were more likely to have low Hb levels at six weeks. There was farm variation in both Hb levels and DG, however, despite this, there was an effect of iron across all farms.

## 1. Introduction

Anaemia caused by iron deficiency has long been reported in dairy calves [1,2]. Iron is primarily sourced from recycled red blood cells at 24 mg/d and diet at 1–2 mg/d [3]. Iron primarily functions as a component of heme, an essential component to haemoglobin’s structure. A deficiency in iron results in reduced haemoglobin (Hb) synthesis, causing reduced red blood cell production and anaemia. New-born calves and piglets are born with finite stores of iron, relying on dietary supplementation once these are used [4]. 

Whole milk contains 0.5 mg of iron/kg (range 0.3 to 0.6 mg/kg) [5]. The most recent dietary guidelines from the National Research Council (2001) suggest that a six-week-old calf growing at 0.8 kg/d, consuming 0.9 kg dry matter/d, needs 150 mg of iron/kg dry matter, so requires 135 mg of iron. To meet this demand from whole milk alone, calves would need to drink 270 L/d. Calves fed whole milk alone are therefore at risk of developing iron deficiency anaemia. Alternative sources of iron include forage, concentrates and small particles of soil ingested whilst grazing [6,7].

Maintaining adequate iron levels in the pre-weaning stages is crucial. Pre-weaned calves reared indoors and fed whole milk have little access to additional iron and are at a higher risk of developing anaemia. Additionally, calves reared under European Union Organic standards are required to be fed whole milk for up to 12 weeks. These extra weeks, coupled with the more expensive, poorly available organic supplements, reduce the chances of alternative dietary sources of iron for these calves. 

This dietary scenario can be likened to indoor fast-growing piglets. There is much evidence to show that they will become iron-deficient if not supplemented. Fast-growing piglets have been found to have lower Hb levels at weaning [4,8] due to higher demand on body iron reserves. Iron has been found to influence development of duodenal cells in piglets’ gastro-intestinal tracts, suggesting an influence on daily liveweight gain (DG) through a direct effect on gut development and immunity [9]. 

The impact on red blood cell production and DG results in piglets routinely being supplemented with iron dextran injections pre-weaning. Piglets can increase their blood volume by 30% in the first week of life if they have adequate iron levels [10].

After the majority of iron is used for Hb production, the remaining 20% circulating is used in many biological processes [11], most commonly immunity. Rajabian et al. found that oxidative stress parameters are increased in iron-deficient calves as iron is used in antioxidative enzymes in erythrocytes. With a decrease in antioxidant capacity, red blood cells are lysed faster, further contributing to the anaemic state. When antioxidant capacities are compromised, this can lead to immunosuppression and potentially increase pre-weaning disease incidence [12]. Bünger et al. looked at pre-weaning disease rates in anaemic and normal calves and showed an increase in incidence of pneumonia in the anaemic calves [13].

There have been several trials conducted on farms where supplementing calves with iron at birth either orally or by injection, has increased haemoglobin levels and growth rates over the first eight weeks of life [14,15]. We are however, lacking evidence on the impact of iron supplementation in whole milk-fed calves in the United Kingdom (UK), particularly the effects on organic calves, possibly creating a barrier for organic bodies to allow iron supplementation to take place. 

The objective of our study therefore was to establish the pre-weaning incidence of iron deficiency anaemia on UK dairy farms feeding whole milk and to investigate the impact of systemic iron supplementation on growth and Hb levels of calves on whole milk diets.

## 2. Materials and Methods 

### 2.1. Study Design 

Sample size calculations revealed that to detect a DG difference of 60 g/d (Standard Deviation (SD) 1.5) as significant at the 5% level with 80% power, the trial required 200 animals, split between control and treatment groups. A convenience sample of six UK farms was recruited from an existing network of known farms and veterinary contacts. Farms were located in Wiltshire, Hampshire, Somerset, Dorset and Devon. Inclusion criteria of farms recruited were: Feeding whole milk pre-weaning (for eight to 12 weeks), rearing own replacement heifers, feeding a starter ration and forage pre-weaning and having a minimum of ten calves eligible to enrol during the trial period. Two farms were all year round (AYR) calving herds and four farms were spring block calving herds. All calves enrolled were female and were Holstein, Friesian and Jersey crosses. Table 1 below describes the enrolled herds in more detail.

Clinically healthy calves were randomly assigned to two groups, treatment with iron injection (INJ) and control, no injection (CON), using the ‘randomiser.org’ app (on the smartphones of the researchers whilst on the farm). One farm had pre-bought electronic ear tags, so a block-randomised list was created for that farm using the same app. INJ calves received five mL iron dextran (Uniferon 20% Injection, Pharmacosmos, Holbæk, Denmark) totalling one gram of iron, administered intramuscularly, into the neck, with an 18 gauge, one-inch needle. Calves had the iron injection between one and ten days of life (average starting age: four days). INJ and CON calves were group-housed together in the same pens on each farm. Every farm had both a treatment and control group to control for any confounding factors on that farm and in total, 237 calves were enrolled between December 2017 and June 2018. 

The calves entered the study at one week old, but this varied between day one and day ten of life (average starting age: four days) after passing a clinical health check. The clinical health check was conducted by a veterinary surgeon before blood sampling. The health check included a full physical exam, including specific anaemia parameters, heart rate and auscultation, respiratory rate, mucous membrane colour and temperature. At this point, calves had blood samples taken using tubes containing ethylenediaminetetraacetic acid (EDTA) from the jugular vein to measure Hb levels and total protein (TP) and were weighed using calibrated electronic scales (Tru test Ezi). The same scales were used on three farms and three farms had their own scales. The calves were weighed again at six weeks old and jugular EDTA blood samples were taken. The calves were weighed only at the age of three months. Each calf ended up with two Hb values and three weights over the pre-weaning period. It was not possible to blind the farmers and researchers to the calves given iron due to statutory farm medicine records. All mortalities were recorded. Veterinary surgeons took the jugular blood samples, blood samples were not taken in relation to feeding times and the calves were weighed by either the vet or farmers. 

Haemoglobin was used as an indirect measure of iron as it is a longer-term measure, widely used [16] and used in the majority of other literature. Within the scientific literature, thresholds ranged from 72.5 g/L to 96 g/L [14,17,18]. Within this study, 72.5 g/L was classified as very low. This is in line with the Welfare of Farmed Animals (England) Regulations 2007 thresholds put in place for veal calves and that described by Volker and Rotermund. Any calves that had Hb levels below 72.5 g/L at the first Hb measurement from both groups were treated with iron dextran and removed from the trial. The threshold of 90 g/L was then used to classify calves as ‘low’ and used as an outcome for suboptimal iron levels [19]. We used the terms ‘low’ and ‘very low’ instead of sub-clinically anaemic and clinically anaemic, because we were only measuring Hb levels and enrolling clinically healthy animals. TP was measured alongside Hb at enrolment, using plasma TP values. The cut off used was 52 g/L [20], with serum and plasma TP levels not differing significantly [21]. The study design did not allow us to evaluate dry cow diet, time of year and breed on Hb levels at the first measurement. EDTA blood samples were collected for Hb values and sent to Scotland’s Rural College laboratory (UKAS accreditation number 2239) in St Boswells, United Kingdom, within 24 h of collection, by recorded delivery first-class post. Samples were not refrigerated. The laboratory used photometric testing for the Hb on a Horiba Vet haematology analyser and Biuret testing for TP on a Randox Imola clinical chemistry analyser. 

### 2.2. Data Analysis 

Data were analysed in SPSS (IBM SPSS Statistics 25). Data are presented as mean, standard deviation (SD) or the Confidence Interval (CI), as appropriate. T-Tests were conducted for individual farms for DG between one to six weeks. Accounting for repeated measures in the calves, we used generalized linear models (GLM) and logistic regression models, with farm as a blocking effect and iron treatment as one of the fixed effects (explanatory variable). We used a multivariate approach to account for confounding factors. Potential confounding factors for DG and Hb were considered to be: age (days) at enrolment, first weight (week one) and failure of passive transfer (TP). External factorsnot taken into consideration, included starter concentrate and milk intakes, environmental temperature and group changes. It is difficult to control for these external factors and it can be assumed as random variation. Some factors will be accounted for by farm being a blocking factor in the statistical evaluation. Differences were considered significant at *p* ≤ 0.05.

### 2.3. Ethical Considerations

The trial was approved by the Royal Veterinary College Clinical Research Ethical Review Board (project number URN 2017 1733-3). This trial was conducted without an Animal Test Certificate. The iron dextran was used under the cascade, as it is not licensed for cattle in the UK, and veterinary surgeons administered the iron. Over the whole study period, no adverse effects of the iron supplementation were seen or reported. 

## 3. Results

Overall, 237 calves were enrolled across six farms (120 calves in the treatment group and 117 calves in control group). Eight calves were excluded as they were below the 72.5 g/L Hb threshold at first measurement, 13 calves died between one to 12 weeks, 10 in the first six weeks and three more between six and 12 weeks. The numbers of calves that died on each farm ranged from zero to six. Nine calves had missing date of birth data and were excluded, two of these were also calves under the 72.5 g/L threshold. The number of calves enrolled on each farm ranged from 18 to 65. 

Data was lost on 12 blood samples due to haemolysis (five), paperwork error (six) and one missed. Two weights of calves were missed at 12 weeks due to calves being moved and not present on the next visit (Appendix A, Table A1). 

The initial weight measurement at one to ten days of age on average was 38.2 kg (5.8 SD), and on average, the CON calves were 0.5 kg (CI 95%: −1.9–0.75 kg) heavier than the INJ calves, but this difference was not significant (*p* = 0.401, n = 222), suggesting the groups were sufficiently randomised. 

### 3.1. Daily Live Weight Gain

#### 3.1.1. One to Six Weeks DG 

**Figure 1:**
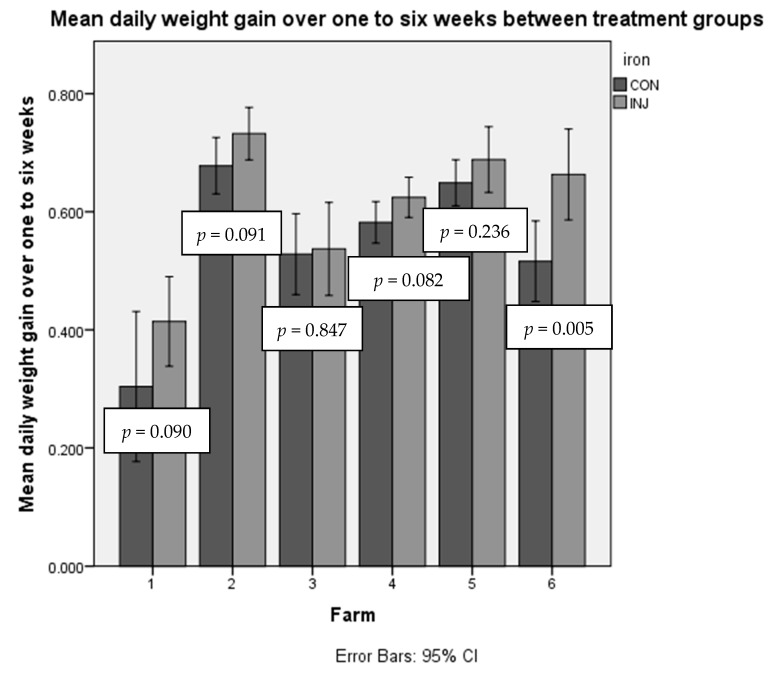
Bar chart showing one- to six-week daily live weight gain (DG) for each farm. Dark grey bars represent CON (control) calves’ average DG on each farm and light grey bars represent INJ (treatment with iron injection) calves’ average DG on each farm. Overall, between all calves, iron increased DG in the INJ group, the difference was 78 g/d (SD 18) (*p* < 0.001). Error bars represent the 95% confidence interval. For number of calves in each group and farm, please see Table A1 in the Appendix A.

The overall DG average across all farms was 566 g/d (SD 170) (Appendix A, Table A2). Figure 1 shows that on all farms, there was a numerical difference between INJ calves and CON calves. The effect of iron injection was 78 g/d (SD 18 g/d, n = 188, 95% CI: 44–112 g/d, *p* < 0.001). This resulted in a 3.3 kg difference on average at six weeks in INJ calves. DG was not affected by birth weight (*p* = 0.789) or age (days) at enrolment (*p* = 0.933). Average TP levels were 70.5 g/L (17.0 SD) and there was no significant correlation between TP and one- to six-week DG (*p* = 0.241). Individual farm differences are detailed in the Appendix A (Table A2, Table A3 and Table A4), including means and individual t-test results. 

#### 3.1.2. Six- to Twelve-Week DG 

The overall average DG across all six farms was 899 g/d (SD 178) for CON and 903 g/d (SD 174) for INJ calves (Appendix A, Table A3). The DG in INJ calves did not differ significantly from that of CON calves (*p* = 0.403, n = 207). DG from six to 12 weeks was not affected by the initial six-week DG (*p* = 0.087), birth weight (*p* = 0.426) and age of enrolment (*p* = 0.153).

#### 3.1.3. One- to Twelve-Week DG 

The overall average DG across all farms was 722 g/d (SD 115) for CON and 750 g/d (SD 108) for INJ calves. The effect of iron was 36 g/d (SD 12) difference (*p* = 0.002, n = 208, 95%CI: 13–58 g/d, Appendix A, Table A4). This resulted in a 3.0 kg difference on average at 12 weeks in INJ calves. The overall one- to 12-week DG was not affected by birth weight (*p* = 0.515) or age of enrolment (*p* = 0.314).

### 3.2. Haemoglobin Levels

The first Hb measurement was comparable across INJ and CON calves (*p* = 0.476, n = 220) suggesting the groups were sufficiently randomized. At the first sampling, eight calves out of 237 (3.4%) were classed as very low (Hb below 72.5 g/L) and 31 calves out of 237 (13.1%) were classed as low (Hb below 90 g/L). At the second Hb measurement at six weeks, four calves out of 203 (2.0%) were classed as very low, 42 out of 203 (20.7%) were classed as low and 121 (60%) calves had a lower Hb level at six weeks than the first week. Hb at the first measurement was also not a significant factor against DG in the first six weeks (*p* = 0.297, n = 188). 

As Table 2 shows, calves in the INJ group at six weeks had a higher average Hb level than the CON group, Hb was 15.3 g/L (SD 1.7), *p* < 0.001, n = 203) higher in the INJ calves. The calves in the CON group dropped, on average, 12.1 g/L (SD 15.1) in the first six weeks of life. The overall was a 15.2 g/L difference (SD 1.9, *p* < 0.001, n = 201) between week one and week six. Hb levels at six weeks were found to be significantly associated with DG at one to six weeks (*p* = 0.05, n = 203) and iron increased both DG and Hb. Once iron was removed from the GLM, Hb at six weeks was no longer associated with DG (*p* = 0.565). The same was the case for one- to 12-week DG (*p* = 0.664, n = 198). 

Calves under the 90 g/L threshold at six weeks (n = 46) were evaluated with a logistic regression model (n = 201). Calves with a higher growth rate (higher than 700 g/d) from one to six weeks were significantly more likely to be under the 90 g/L threshold (Odds Ratio (OR) 5.0, 95% CI:1.7–14.2, *p* = 0.003). CON group calves were also more likely to be under the 90 g/L threshold (OR 19.7, 95% CI: 6.5–59.6 , *p* < 0.001) and if calves had a high Hb (higher than 108 g/L) at the first measurement, they were less likely to be under the 90 g/L threshold (OR 0.3, 95% CI: 0.1–0.7, *p* = 0.005).

## 4. Discussion

This is one of the first studies in the UK investigating the effect of iron supplementation on whole milk-fed calves. This study focused on the first 12 weeks of life and found an average increase in DG of 78 g/d in the first six weeks to calves given a supplementary iron injection. Even though there were variations in calf-rearing practices, the fact that there was a numerical DG difference on all farms highlights that iron had an effect, despite varying environmental factors. Individual farm calf-rearing practices were not measured, except for nutrition (whole milk for eight to 12 weeks). It was accepted that there would be some farm variation in DG due to slightly different calf-rearing standards on the different farms, and that this should mostly be dealt with using farm as a blocking factor in GLM models. The reason for this was that the focus was on recruiting larger numbers needed for this study, to evaluate iron supplementation at a herd level. It can be confidently said that iron makes a difference to DG in the first six weeks, but the magnitude of effect seems to be farm-specific, as there is a notable difference between farm DG variation. 

Increased DG in the pre-weaning period has been shown to improve first lactation success in yield and mammary tissue development [22,23,24]. With increasing volumes of milk fed and with organic farms stipulated to not wean until 12 weeks, iron supplementation may find a place in maintaining these high DGs. Iron also plays a role in immunity and oxidative stress [3,12,25,26]. Rajabian et al. [12] showed that erythrocytes had an impaired antioxidant defence system in calves with iron deficiency, and these calves had increased oxidative stress parameters. Antioxidant defences play an important role in tissue defences to free radical damage and on a bigger scale, defence against calf pathogens as a whole, for example diarrhoea and damage to the gut wall. A reduction in immunity could be a contributing factor to a lowered DG in CON calves, due to a higher pre-weaning disease incidence in iron-deficient calves. As this was a convenience sample of farms, pre-weaning health and treatment data quality was not taken into account and was of inadequate quality to use on some of the farms. We were unable to compare disease incidence between groups, and this is an area that needs to be researched in more detail.

The DG difference between INJ and CON groups was not significant from six to 12 weeks old. When both six-week blocks were looked at together, iron injection was a significant factor in an increased DG, however, this mainly represents the first six weeks of life. The duration of action of the iron dextran is unknown in calves, as it is such a relatively small dose compared to piglets, so potentially it may have dropped to below therapeutic levels. The dose usually given in piglets is considerably higher than the chosen dose in calves (200 mg/kg versus 25 mg/kg), to allow for the much faster relative DG. Piglets also commonly receive two injections of iron dextran, so, potentially, calves could require another dose to see a continued affect. Bostedt found the dose of 1000 mg per calf to have the best effect on Hb levels, once raised over this to 1500 mg, the end Hb levels at 43 days were the same [27]. Other previous studies have used oral supplementation, which is now known to be poorly absorbed, ranging from 40 mg/d to 400 mg/d [1,14,28,29] to varying effects. Only Mohri et al. used injectable iron dextran at 1000 mg as a one-off injection and found a 4.8 kg weight increase in end weights in their treated groups [15]. Despite better absorption, iron dextran injections are off license in cattle and can cause severe reactions in humans, so test doses are advisable [30]. However, the product used was a low molecular weight iron dextran, which has been shown to have a reduced risk, and no adverse reactions were observed during our study. 

There were a considerable number of calves classified as having low Hb levels (under the 90 g/L threshold) at both one (13.1%) and six weeks (20.7%) old. Iron dextran injection significantly increased Hb levels over the first six weeks, with 40 calves still under 90 g/L in the CON group at six weeks. The difference between the average Hb levels at six weeks in the INJ and CON calves was also statistically significant. Ramin et al. recorded calves with the lowest levels of Hb around three months old (<74 g/L) [7]. Other historic research on oral iron supplementation found an increase in Hb levels from 54 to 80 g/L [31], 71 to 105 g/L [32] and the lowest recorded levels at 43.8 g/L at 12 weeks in control calves [33].

Hb levels are used as a guideline for measuring iron in much of the previously published literature, in spite of known limitations, such as variations due to haemoconcentration or haemodilution. In this trial, only healthy animals were included. We understand there may be some individual variation, but this background variation should be the same in both treatment groups and as we were focusing on the herd level effect of iron. On the six farms analysed, both the DG difference and the Hb levels varied drastically. This was supported by the fact that Hb at six weeks was only significant when iron was included in the model, without iron, Hb at six weeks was not a significant factor for DG in the first six weeks. In this study, average Hb levels on individual farms were not predicting the size of the benefit of iron supplementation for that farm. 

Higher starting levels of Hb were significantly associated with calves being over 90 g/L at six weeks, so it was questioned if a dry cow diet would influence Hb levels transferred to the foetus. However, it is unlikely that dry cow diets would affect neonatal calf Hb levels. Hibbs et al. found that dam and calf Hb levels were not correlated at birth [2] and more recently, Potthoff found that there is little iron transfer between dam and calf when in-utero, so direct administration is still needed once the calf is born [34]. Due to the very low levels of iron in milk, the level of supplementation to the dam would have to be very high to have any effect. Additionally, once cattle are ruminating, iron absorption efficiency is lowered due to forage providing the amounts needed, to prevent the animal from toxicity [6].

The calves in our study have all been reared on conventional and organic UK dairy farms as replacement heifers. These Hb levels potentially indicate that these calves had inadequate intakes of iron during the first six weeks, resulting in our result of lower DG in CON calves. Interestingly, our finding that calves under 90 g/L at six weeks were significantly more likely to be the faster growing calves, shows that there is a high demand on the body’s iron reserves in this time period. This finding echoes similar work on piglets: Perri et al. found that larger piglets at weaning had lower Hb levels [4]. Within the same model, the iron injection was also significant, meaning that supplementation in these calves did mitigate the risk of them being under 90 g/L at six weeks. 

## 5. Conclusions

The effect of increased DG pre-weaning is well known to have long-term benefits. This data has shown that calves on whole milk diets injected with iron have a significantly increased DG in the first six weeks. Iron injection also resulted in significantly higher Hb levels at six weeks and prevented Hb levels from dropping in the first six weeks of life. Faster growing calves are more likely to be under the 90 g/L threshold at six weeks, and we have shown that this is mitigated with systemic iron supplementation.

There was farm variation in treatment response for both Hb levels and DG. However, there was an overall effect of iron supplementation, suggesting we can generalise the relevance across farms. With current industry recommendations to feed higher volumes of milk for longer, there may be a place for systemic iron supplementation in whole milk-fed calves. Future work needs to be carried out to establish the link between early iron deficiency and long-term DG in calves as well as investigating the link to pre-weaning disease incidence. 

## Figures and Tables

**Table 1:** Details of farms enrolled, volume of milk and ration fed, calving pattern, number of calves enrolled and starting haemoglobin levels and bodyweight values.

Farm Factors	Farm 1	Farm 2	Farm 3	Farm 4	Farm 5	Farm 6
Volume of whole milk fed per day (fed via bucket with teat)	6 L	6 L	5 L	5 L	6 L	6 L
Starter ration	Yes, from birth	Yes, from birth	Yes, from week 1	Yes, from 2–3 weeks	Yes, from birth	Yes, from birth
Calving pattern	All year round	Spring	All year round	Spring	Spring	Spring
Number of calves enrolled	28	38	18	65	40	48
Average bodyweight at first weighing (SD) *	39.3 (4.4)	43.7 (7.9)	42.7 (3.6)	34.7 (4.3)	38.7 (4.5)	37.1 (4.7)
Average haemoglobin (g/L) levels at first measurement (SD)	114.0 (20.0)	105.0 (16.3)	105.6 (17.1)	110.3 (13.4)	108.6 (12.9)	102.9 (13.7)

* Standard deviation.

**Table 2:** Average haemoglobin (Hb) values at six weeks, Hb difference between the first Hb measurement and Hb measurement at six weeks and number of ‘low’ calves in both treatment (INJ) and control (CON) calves on each farm. These are mathematical means reported.

Farm	Average Hb Six Weeks (g/L) (SD)			Average Change in Hb Level One to Six Weeks (g/L) (SD)			Number of Calves with HB Levels under 90 g/L at Six Weeks	
	INJ	CON	Difference between Groups	INJ	CON	Difference between Groups	INJ	CON
1	120.9 (14.6)	106.9 (10.0)	14	9.8 (18.0)	−6.2 (20.9)	16	0	0
2	108.1 (6.4)	87.9 (12.2)	18.9	5.2 (11.3)	−16.8 (10.7)	19.3	0	8 (2) *
3	105.0 (10.4)	83.5 (5.2)	19.8	0.4 (5.7)	−12.3 (6.8)	11.8	0	4
4	109.1 (11.5)	95.5 (10.8)	13.6	−0.5 (11.5)	−15.4 (12.6)	14.9	2	13
5	113.6 (13.9)	95.9 (13.1)	17.7	3.9 (15.5)	−11.2 (12.9)	15.1	2	4 (1) *
6	108.0 (11.9)	95.6 (15.6)	12.4	4.4 (12.6)	−7.3 (19.0)	11.7	2	7 (1) *
Overall average	110.7 (12.4)	94.9 (13.2)	15.3 (1.7)	3.4 (13.3)	−12.1 (15.1)	15.2 (1.9)	6 total	40 total

* Calves under 72.5 g/L.

## Appendix A

**Table A1:** Overall numbers of calves enrolled on each farm and in each treatment group, control (CON) and treatment (INJ) number of calves lost pre-weaning, number of calves excluded, and number of blood samples lost, and weights missed. Mathematical means are presented.

Farm	Number of Calves Enrolled	Number of Calves in CON Group	Number of Calves in INJ Group	Dead One to Six Weeks	Dead One to 12 Weeks	Missed Bloods or Weights	Excluded for no Date of Birth	Excluded Hb under 72.5 g/L at Week One
1	28	13	15	6	1	0	1	0
2	38	19	19	1	1	1	6, one is also under 72.5 g/L	3
3	18	8	10	0	0	5	2, one is also under 72.5 g/L	3
4	65	33	32	1	1	3	0	0
5	40	19	21	2	0	4	0	1
6	48	25	23	0	0	1	0	1
Total	237	117	120	10	3	14	9	8

**Table A2:** Daily live weight gain differences on each farm from one to six weeks old. Mathematical means are presented.

Farm	Treatment Group (g/d) (SD)	Control Group (g/d) (SD)	Difference (g/d)	*p*-Value from Independent T-Test for Each Farm	Overall Daily Live Weight Gain (g/d) (SD)
1	414 (126)	304 (165)	110	0.090	369 (150)
2	732 (74)	678 (90)	54	0.091	702 (86)
3	537 (94)	528 (65)	9	0.847	533 (80)
4	624 (93)	582 (98)	42	0.082	603 (97)
5	688 (116)	649 (79)	39	0.236	669 (100)
6	663 (178)	516 (162)	147	0.005	588 (184)
Average	625 (151)	566 (170)	59		

**Table A3:** Daily live weight gain differences on each farm from six to 12 weeks old. Mathematical means are presented.

Farm	Treatment Group (g/d) (SD)	Control Group (g/d) (SD)	Difference (g/d)	Overall Daily Live Weight Gain (g/d) (SD)
1	861 (178)	846 (75)	15	855 (145)
2	980 (142)	1014 (174)	–34	998 (158)
3	1017 (139)	906 (147)	111	970 (148)
4	1017 (134)	980 (108)	37	998 (122)
5	872 (107)	937 (137)	–65	904 (126)
6	725 (125)	708 (167)	17	717 (147)
Average	903 (174)	899 (178)	4	

**Table A4:** Daily live weight gain differences on each farm from one to 12 weeks old. Mathematical means are presented.

Farm	Treatment Group (g/d) (SD)	Control Group (g/d) (SD)	Difference (g/d)	Overall Daily Live Weight Gain (g/d) (SD)
1	632 (124)	591 (60)	41	617 (105)
2	865 (73)	852 (93)	13	858 (83)
3	776 (84)	715 (85)	61	750 (87)
4	768 (80)	730 (81)	38	749 (82)
5	780 (99)	792 (86)	–12	786 (91)
6	696 (72)	621 (72)	75	658 (81)
Average	750 (108)	722 (115)	28	

## References

[1] Knoop C., Krauss W., Washburn R. (1935). The Development of Nutritional Anemia in Dairy Calves. J. Dairy Sci..

[2] Hibbs J., Conrad H., Vandersall J., Gale C. (1963). Occurrence of Iron Deficiency Anemia in Dairy Calves at Birth and Its Alleviation by Iron Dextran Injection. J. Dairy Sci..

[3] Cherayil B.J. (2011). The role of iron in the immune response to bacterial infection. Immunol. Res..

[4] Perri A.M., Friendship R.M., Harding J.C.S., O’Sullivan T.L. (2016). An investigation of iron deficiency and anemia in piglets and the effect of iron status at weaning on post-weaning performance. J. Swine Health Prod..

[5] Hunt C., Nielsen F., McSweeney P., Fox P.F. (2009). Nutritional Aspects of Minerals in Bovine and Human Milks. Advanced Dairy Chemistry.

[6] National Research Council (2001). Minerals. Nutrient Requirements of Dairy Cattle.

[7] Ramin A.G., Asri-Rezaei S., Paya K., Eftekhari Z., Jelodary M., Akbari H., Ramin S. (2014). Evaluation of Anemia in Calves up to 4 Months of Age in Holstein Dairy Herds. İstanbul Üniversitesi Veteriner Fakültesi Dergisi.

[8] Bhattarai S., Nielsen J. (2015). Early indicators of iron deficiency in large piglets at weaning. J. Swine Health Prod..

[9] Pu Y., Li S., Xiong H., Zhang X., Wang Y., Du H.-H. (2018). Iron Promotes Intestinal Development in Neonatal Piglets. Nutrients.

[10] Svoboda M., Drabek J. (2005). Iron deficiency in suckling piglets: Ethiology, clinical aspects and diagnosis. Folia Vet..

[11] Beard J.L. (2001). Iron biology in immune function, muscle metabolism and neuronal functioning. J. Nutr..

[12] Rajabian F., Mohri M., Heidarpour M. (2017). Relationships between oxidative stress, haematology and iron profile in anaemic and non-anaemic calves. Veter. Rec..

[13] Bünger U., Schmoldt P., Ponge J. (1986). Oral and parenteral control of iron deficit in relation to the course of diseases in milk fed calves originating from different farms. Monatsh. Veterinarmed..

[14] Volker H., Rotermund L. (2000). The possibilities of oral iron supply in calves to maintain their health status. Ger. Vet. Wkly..

[15] Mohri M., Poorsina S., Sedaghat R. (2009). Effects of Parenteral Supply of Iron on RBC Parameters, Performance, and Health in Neonatal Dairy Calves. Boil. Trace Element Res..

[16] Jones M., Allison R.W. (2007). Evaluation of the Ruminant Complete Blood Cell Count. Veter. Clin. North Am. Food Anim. Pr..

[17] RSPCA (2018). RSPCA Welfare Standards for Dairy Cattle. https://www.ciwf.org.uk/media/5235182/Statistics-Dairy-cows.pdf.

[18] Dillane P., Krump L., Kennedy A., Sayers R.G., Sayers G.P. (2018). Establishing blood gas ranges in healthy bovine neonates differentiated by age, sex, and breed type. J. Dairy Sci..

[19] UK legislation The Welfare of Farmed Animals (England) Regulations 2007, Animal Welfare Act 2006. http://www.legislation.gov.uk/uksi/2007/2078/schedule/6/made.

[20] Macfarlane J.A., Grove-White D.H., Royal M.D., Smith R. (2014). Use of plasma samples to assess passive transfer in calves using refractometry: Comparison with serum and clinical cut-off point. Veter. Rec..

[21] Elsohaby I., McClure J., Waite L., Cameron M., Heider L., Keefe G. (2019). Using serum and plasma samples to assess failure of transfer of passive immunity in dairy calves. J. Dairy Sci..

[22] Brickell J., Bourne N., McGowan M., Wathes D.C. (2009). Effect of growth and development during the rearing period on the subsequent fertility of nulliparous Holstein-Friesian heifers. Theriogenology.

[23] Soberón F., Raffrenato E., Everett R., Van Amburgh M. (2012). Preweaning milk replacer intake and effects on long-term productivity of dairy calves. J. Dairy Sci..

[24] Wathes D.C., Pollott G.E., Johnson K.F., Richardson H., Cooke J.S. (2014). Heifer fertility and carry over consequences for life time production in dairy and beef cattle. Anim..

[25] Cherayil B.J. (2010). Iron and Immunity: Immunological Consequences of Iron Deficiency and Overload. Arch. Immunol. Ther. Exp..

[26] Cassat J.E., Skaar E.P. (2013). Iron in infection and immunity. Cell Host Microbe.

[27] Bostedt H., Hospes R., Wehrend A., Schramel P. (2000). Effects of parenteral administration of iron preparations in the early development of calves. Vet. Rev..

[28] Kume S.-I., Tanabe S. (1996). Effect of Supplemental Lactoferrin with Ferrous Iron on Iron Status of Newborn Calves. J. Dairy Sci..

[29] Mohri M., Sarrafzadeh F., Seifi H. (2006). Effect of oral iron supplementation on hematocrit, live weight gain and health of neonatal dairy calves. Iranian J. Vet. Med. Res..

[30] BNF (2019). Iron Dextran, Institute for Health and Care Excellence, National. https://bnf.nice.org.uk/drug/iron-dextran.html.

[31] Matrone G., Conley C., Wise G., Waugh R. (1957). A Study of Iron and Copper Requirements of Dairy Calves. J. Dairy Sci..

[32] Thomas J., Okamoto M., Jacobson W., Moore L. (1954). A Study of Hemoglobin Levels in the Blood of Young Dairy Calves and the Alleviation of Anemia by Iron. J. Dairy Sci..

[33] Blaxter K.L., Sharman G.A.M., Macdonald A.M. (1957). Iron-deficiency anaemia in calves. Br. J. Nutr..

[34] Potthoff B. (2011). Influence of Oral Iron Supplements (Iron Amino Acid Chelate) in the Dry Period on Parameters of the Iron Metabolism of Cows and Their Newborn Calves. Dissertation Thesis.

